# Shenfu injection: a review of pharmacological effects on cardiovascular diseases

**DOI:** 10.3389/fphar.2024.1279584

**Published:** 2024-02-14

**Authors:** Fei-Fei Xu, Xiao-Fang Xie, Hai-Yan Hu, Rong-Sheng Tong, Cheng Peng

**Affiliations:** ^1^ State Key Laboratory of Southwestern Chinese Medicine Resources, Chengdu University of Traditional Chinese Medicine, Chengdu, China; ^2^ Department of Pharmacy, Personalized Drug Therapy Key Laboratory of Sichuan Province, Sichuan Academy of Medical Sciences and Sichuan Provincial People’s Hospital, School of Medicine, University of Electronic Science and Technology of China, Chengdu, China; ^3^ Sichuan Nursing Vocational College, Chengdu, China

**Keywords:** Shenfu injection, cardiovascular disease, heart failure, pharmacology, TCM (traditional Chinese medicine), clinical trial

## Abstract

Shenfu injection (SFI), composed of ginseng and aconite, is a Chinese patent developed from the classic traditional prescription Shenfu Decoction created more than 700 years ago. SFI has been widely used in China for over 30 years for treating cardiovascular diseases. The main components in it include ginsenosides and aconitum alkaloids. In recent years, the role of SFI in the treatment of cardiovascular diseases has attracted much attention. The pharmacological effects and therapeutic applications of SFI in cardiovascular diseases are summarized here, highlighting pharmacological features and potential mechanisms developments, confirming that SFI can play a role in multiple ways and is a promising drug for treating cardiovascular diseases.

## 1 Introduction

Cardiovascular diseases (CVDs) remain the predominant cause of mortality and morbidity worldwide over the past 20 years, including atherosclerosis, coronary heart disease, arrhythmia, hypertension, cardiomyopathy, stroke and heart failure (Parikh et al., 2018; Benjamin et al., 2019; Feng et al., 2019; Makhmudova et al., 2021). According to the World Health Organization (WHO) Report 2021, noncommunicable diseases (NCDs) kill more than 40 million people every year, and CVDs are the world’s leading cause of death, accounting for almost one in three of all reported deaths globally. Data from the World Heart Report 2023 shows that 20.5 million people died from CVDs in 2021 (Mariappan et al., 2023). CVDs are caused by a variety of pathological factors, such as atherosclerosis, hypertension, hyperlipidemia, diabetes mellitus and so on, associated with energy metabolism disorder, mitochondrial structure abnormality, oxidative stress injury, cardiomyocyte apoptosis, inflammatory reaction, but the specific pathogenesis has not yet been fully elucidated (Parikh et al., 2018; Benjamin et al., 2019; Feng et al., 2019; Makhmudova et al., 2021). Based on the complex pathophysiologcial mechanisms, there are numerous drugs recommended for the treatment of CVDs, including angiotensin-converting enzyme inhibitors, angiotensin receptor antagonists, β-receptor antagonists, vasodilators, diuretics, α-receptor antagonists, positive inotropes, lipid-lowering drugs, antiarrhythmics, calcium channel blockers, *etc.* However, their potential serious adverse effects caused by these drug, such as hyperkalemia, cardiac depression, and electrolyte disturbance, cannot be ignored (Alhawassi et al., 2018; Núñez-Acevedo et al., 2018; Pall et al., 2021). Therefore, folk medicine is widely used to treat CVDs, among which traditional Chinese medicine (TCM) is well known in the world. Along with the long history of development for TCM, some classic recipes for the treatment of CVDs have been used in the clinic since then. Zhigancao Decoction, originated from *Treatise on Febrile Diseases* in the Eastern Han Dynasty (25–280 AD), is composed of Glycyrrhiza uralensis Fisch [Leguminosae; Glycyrrhizae Radix et Rhizoma], Zingiber officinale Rosc [Zingiberaceae; Zingiberis Rhizoma Recens], Cinnamomum cassia Presl [Lauraceae; Cinnamomi Ramulus], Panax ginseng C.A.Mey [Araliaceae; Ginseng Radix et Rhizoma Rubra], Rehmannia glutinosa Libosch [Scrpophulariaceae; Rehmanniae Radix], *Equus asinus* L [Equidae; Asini Corii Colla], Ophiopogon japonicus (L.f) Ker-Gawl [Liliaceae; Ophiopogonis Radix], Cannabis sativa L [Moraceae; Cannabis Fructus], Ziziphus jujuba Mill [Rhamnaceae; Jujubae Fructus], and used to treat arrhythmia and heart failure (Xiong, 2019; Zhang N. et al., 2021; Wu et al., 2021; Yang Y. et al., 2022). Xuefu Zhuyu Decoction, recorded in the classic *Yi Lin Gai Cuo* in the Qing dynasty (1830 AD), composed of eleven commonly used herbs, including Prunus persica (L.) Batsch [Rosaceae; Persicae Semen], Carthamus tinctorius L [Compositae; Carthami Flos], Angelica sinensis (Oliv.) Diels [Umbelliferae; Angelicae Sinensis Radix], Rehmannia glutinosa Libosch [Scrpophulariaceae; Rehmanniae Radix], Achyranthes bidentata Bl [Amaranthaceae; Achyranthis Bidentatae Radix], Ligusticum chuanxiong Hort [Umbelliferae; Chuanxiong Rhizoma], Platycodon grandiflorum (Jacq.) A. DC [Campanulaceae; Platycodonis Radix], Paeonia lactiflora Pall [Ranunculaceae; Paeoniae Radix Rubra], Citrus aurantium L [Rutaceae; Aurantii Fructus], Glycyrrhiza uralensis Fisch [Leguminosae; Glycyrrhizae Radix et Rhizoma], Bupleurum chinense DC [Umbelliferae; Bupleuri Radix], is used to treat hyperlipidemia and coronary heart disease (Wang and Qiu, 2019; Zhang S. et al., 2021; Yang et al., 2023). Zhenwu Decoction, was firstly recorded in *Treatise on Febrile Diseases*. It inculdes five herbs: Poria cocos (Schw.) Wolf [Polyporaceae; Poria], Paeonia lactiflora Pall [Ranunculaceae; Paeoniae Radix Alba], Zingiber officinale Rosc [Zingiberaceae; Zingiberis Rhizoma Recens], Aconitum carmichaelii Debx [Ranunculaceae; Aconiti Lateralis Radix Praeparata], Atractylodes macrocephala Koidz [Compositae; Atractylodis Macrocephalae Rhizoma], which is applied to treat chronic heart failure (Tang et al., 2018; Han et al., 2022). It is believed in TCM that CVDs is related to the imbalance of Qi, Xue, Yin and Yang in the human body. When Qi and Yang is insufficient, CVDs are prone to occur.

**FIGURE 1:**
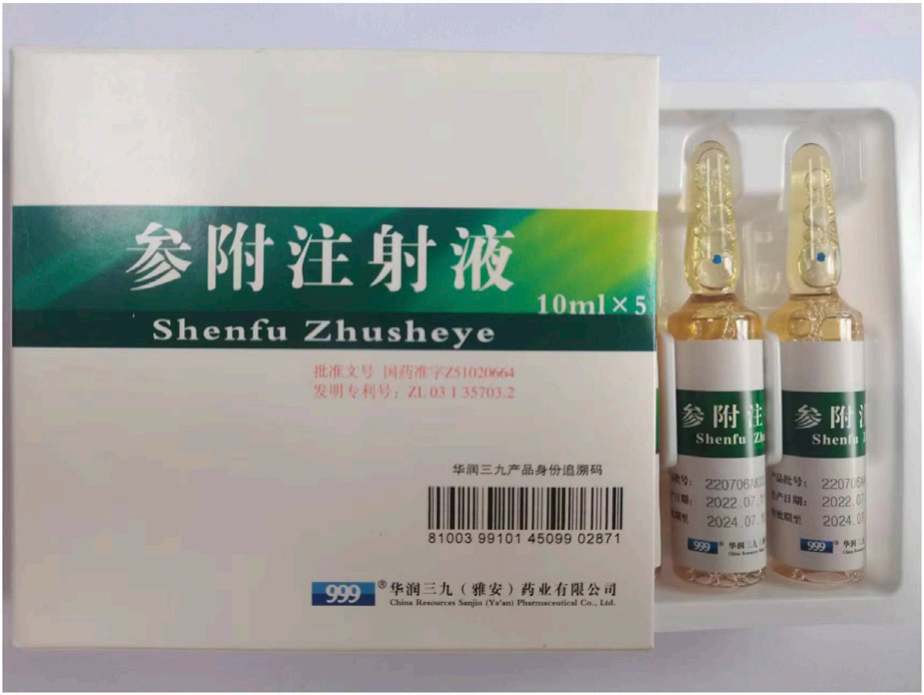
Shenfu injection.

SFI (Figure 1) is widely used in China to treat numerous ailments, including shock (Zhang X. et al., 2020; Wang et al., 2022; Zhang and Li, 2023), pulmonary fibrosis (Liu et al., 2021), sepsis (Luo et al., 2021; Li X. et al., 2022; Xu et al., 2022), pneumonia (Niu et al., 2021; Shi et al., 2022), cancer (Gao and Zhang, 2023; Wen et al., 2023), cercerebral infarction (Zhou et al., 2020), CVDs, and has shown promising results. With development of pharmacological research, SFI has been identified as an effective drug for the treatment of CVDs.This paper reviews the latest reports in the past 20 years (2003–2022) from PubMed, Web of Science, and National Knowledge Infrastructure (CNKI) using the keywords “Shenfu injection” and “cardiovascular diseases”. The pharmacological action and therapeutic application of SFI in treating CVDs were discussed, and its pharmacological characteristics and potential mechanism was emphasized.

## 2 SFI -basic characteristics and history of use

**FIGURE 2:**
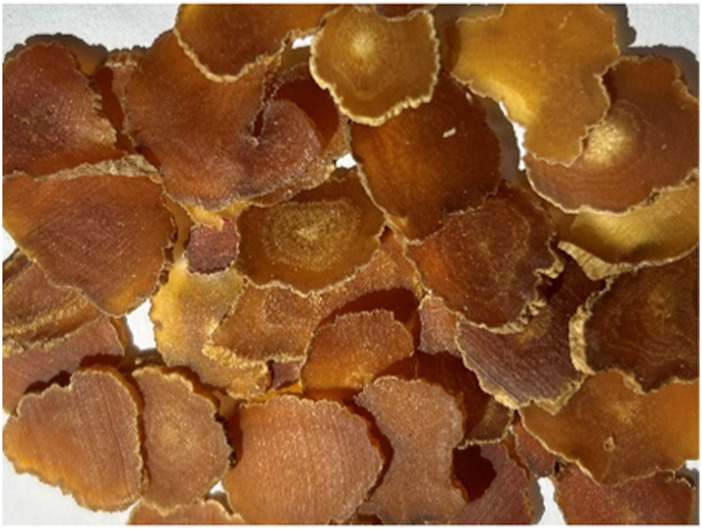
Ginseng Radix et Rhizoma Rubra.

**FIGURE 3:**
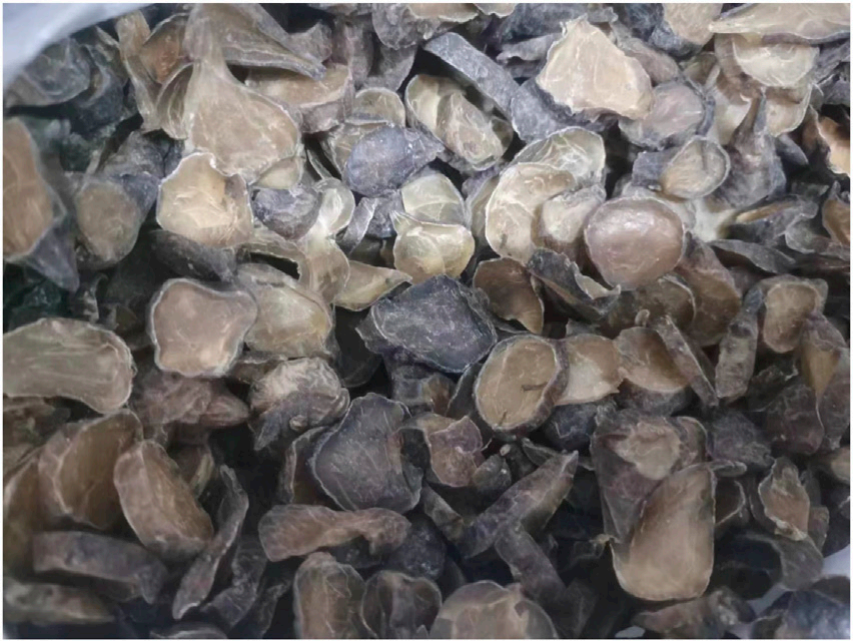
Aconiti lateralis radix praeparata.

SFI is a commonly used traditional Chinese medicine injection that has been used in clinical for over 30 years (Liu et al., 2021). It originated from the traditional Chinese classical formula “Shenfu Decoction", which was first recorded in *Yan’s Prescriptions for Rescuing Lives* in the Song Dynasty (1253 AD). SFI is composed of Panax ginseng C.A.Mey [Araliaceae; Ginseng radix et rhizoma rubra] (RG) (Figure 2) and Aconitum carmichaelii Debx [Ranunculaceae; Aconiti lateralis radix praeparata] (RA) (Figure 3), which has the function of restoring Yang and invigorating Qi (Pei et al., 2021; Zhou et al., 2022). The existing studies reported that RG can be used to treat coronary heart disease and atherosclerosis by reducing blood lipid levels and improving inflammation (Hernández-García et al., 2019; Lu et al., 2019; Im, 2020). Additionally, it can inhibit arrhythmia by affecting the ion channels, such as activating potassium channel while blocking calcium channel and sodium current (Liu Z. et al., 2019; Gou et al., 2020). Furthermore, by ameliorating mitochondrial function and reducing oxidative damage in cardiomyocytes, it can prevent ventricular remodeling and heart failure. Moreover, it has the potential to improve the function of vascular endothelial cells, thereby lowering blood pressure (Yang F. et al., 2022; Liu et al., 2022). Meanwhile, RA has cardiotonic effects by accelerating β-adrenergic receptor synthesis (Tong et al., 2021), has anti-inflammatory effects through the Toll-like receptor4/Nuclear factor κB (TLR4/NF-κB) pathway (Yan et al., 2020), and has anti-arrhythmic effects (Wang et al., 2023). SFI, composed of RA and RG, is a common drug for the treatment of CVDs.

Modern chemical studies have shown that SFI mainly contains ginsenosides, aconite alkaloids, organic acids, nucleosides, amino acids and other components (Song et al., 2015). Ginsenosides and aconite alkaloids are the main active components of SFI. The content of ginsenosides is 676–742 μg/mL, and the content of aconite alkaloids is 3–7 μg/mL (Yang et al., 2014; Ge et al., 2015; Song et al., 2015). It is known that aconite has certain toxicity, and the use of RG and RA in combination can achieve the effect of potentiation and detoxification. Ginsenosides can promote the metabolism of the toxic component aconitine, prolong the elimination half-life of active ingredients such as hypaconitine, benzoylmesaconine and songorine, and significantly increase the *in vivo* exposure of active ingredients. At the same time, some studies have found that ginseng can inhibit the ion disorders, toxicity in calcineurin-nuclear factor of activated T cells (CaN-NFAT3) pathway and inhibition of the cytochrome P450 2J3 (CYP2J3) expression caused by aconitine, and enhance the antioxidant effect of myocardial cells (Liu et al., 2020; Yang et al., 2021; Chen Z. Y. et al., 2022; Bao et al., 2023). Therefore, the compatibility of aconite and ginseng has the effect of ‘reducing toxicity and increasing efficiency’.

## 3 Bioavailability and metabolism of SFI

**FIGURE 4:**
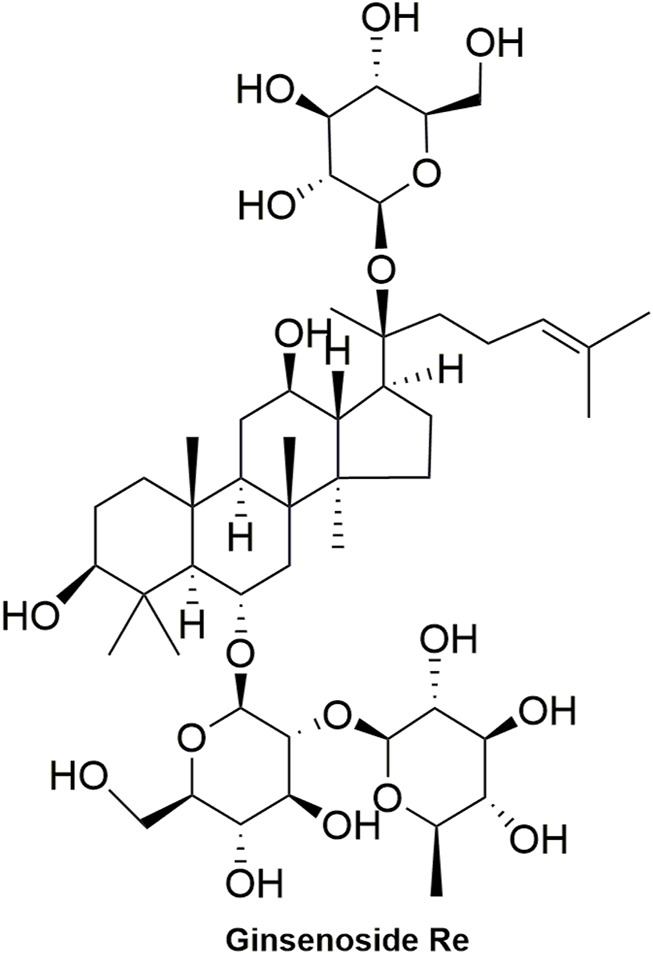
Structural formula of ginsenoside Re.

**FIGURE 5:**
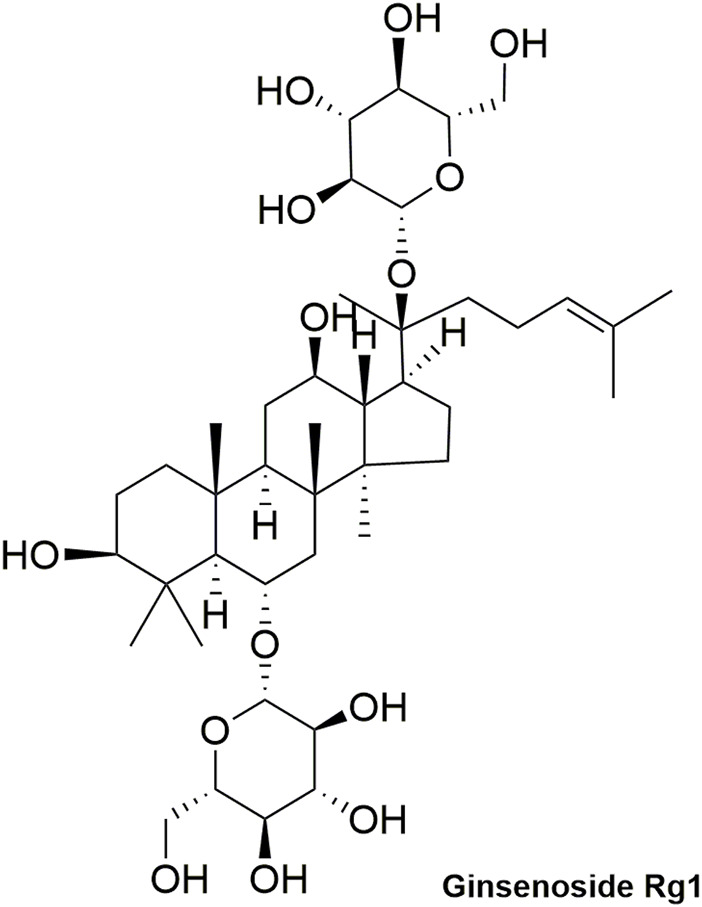
Structural formula of ginsenoside Rg1.

**FIGURE 6:**
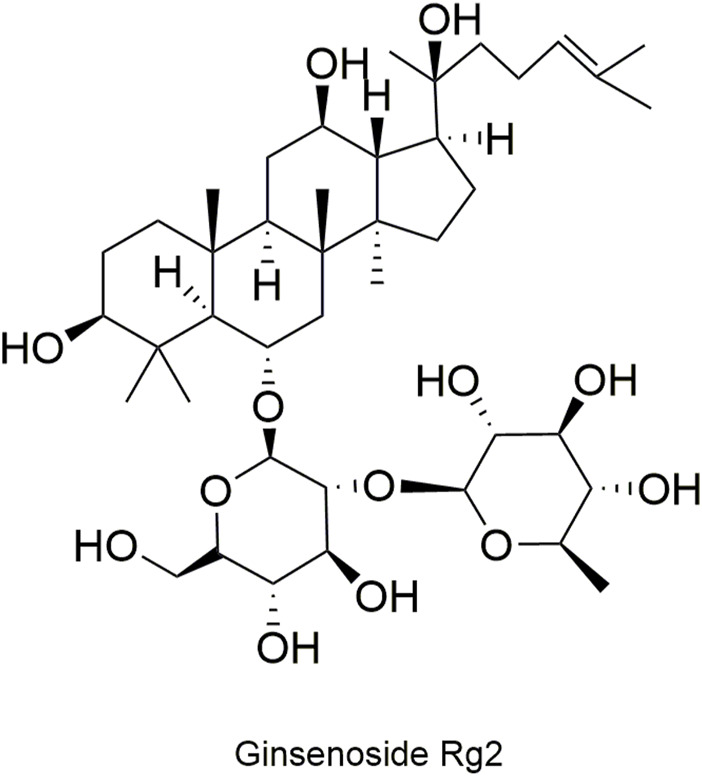
Structural formula of ginsenoside Rg2.

**FIGURE 7:**
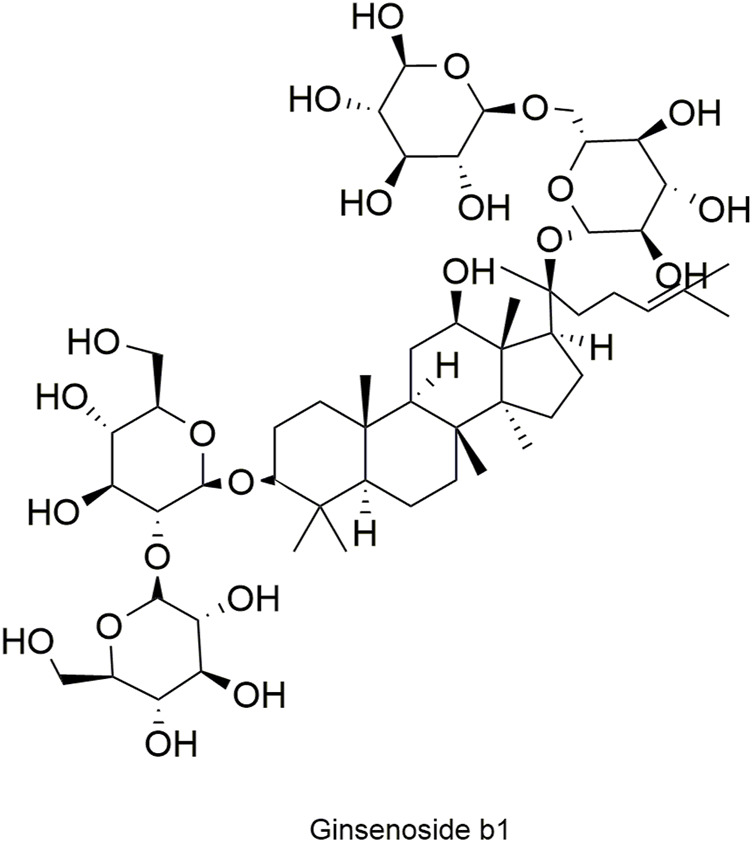
Structural formula of ginsenoside Rb1.

**FIGURE 8:**
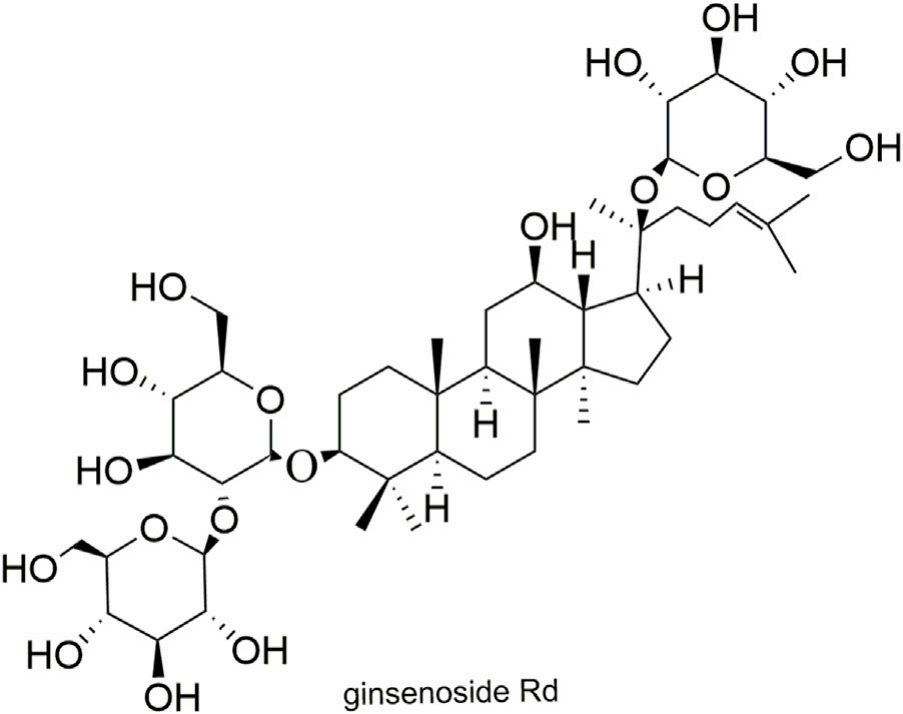
Structural formula of ginsenoside Rd.

**FIGURE 9:**
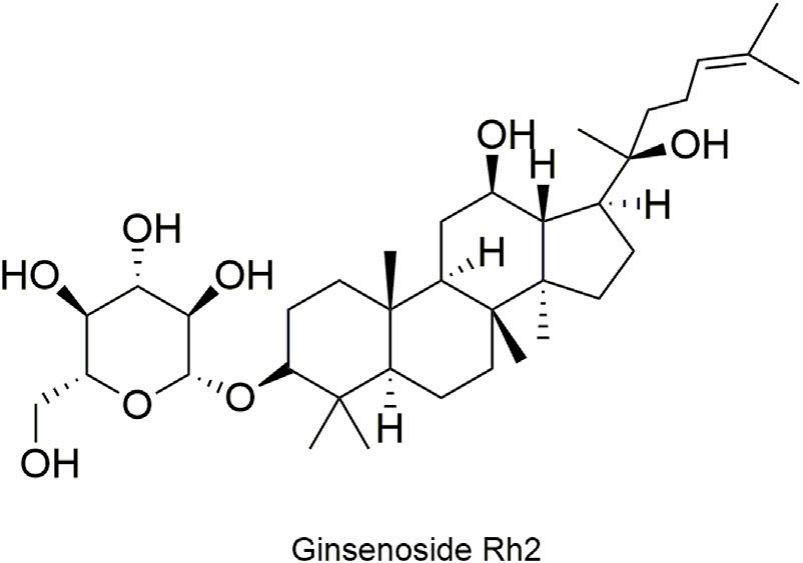
Structural formula of ginsenoside Rh2.

**FIGURE 10:**
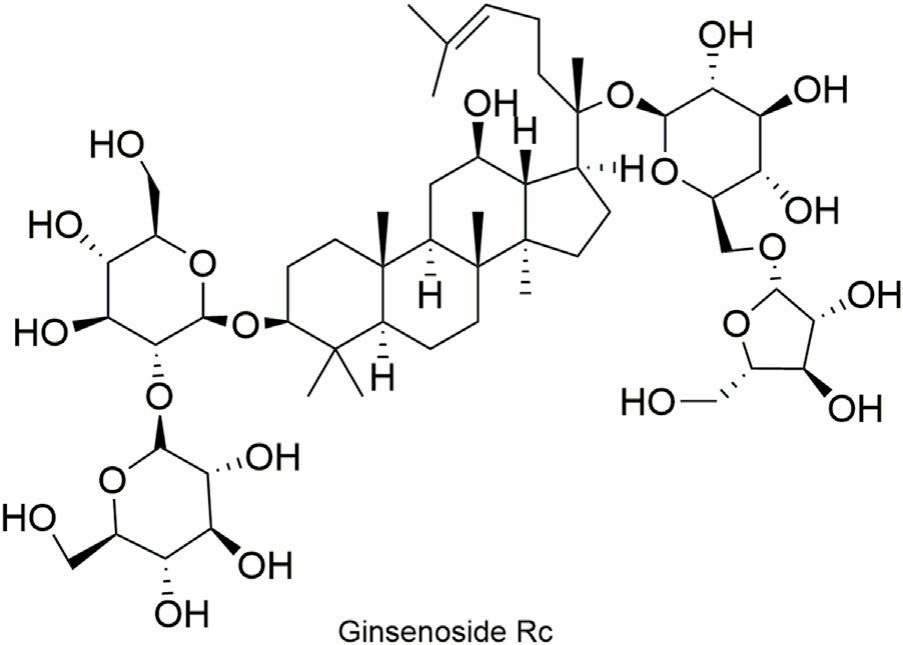
Structural formula of ginsenoside Rc.

**FIGURE 11:**
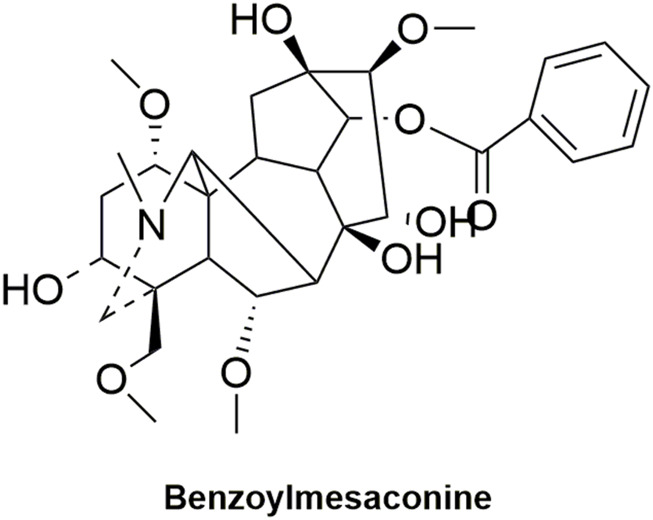
Structural formula of benzoylmesaconine.

**FIGURE 12:**
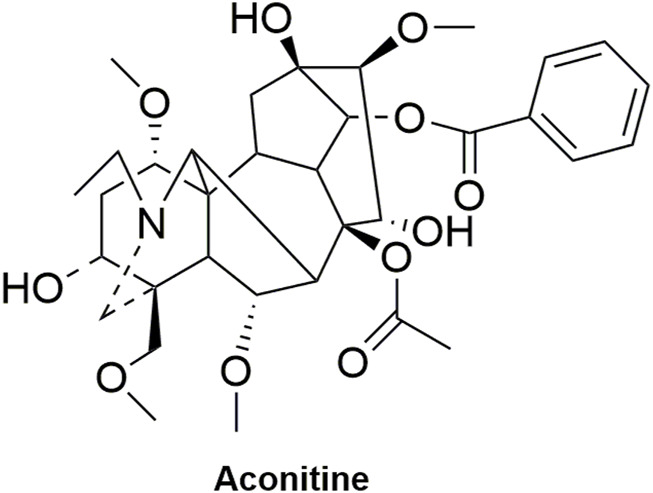
Structural formula of aconitine.

Pharmacokinetic data of rodents show that aconitum alkaloids can be rapidly eliminated after intravenous injection of SFI. Protopanaxatriol (PPT) ginsenosides such as ginsenoside Re (Figure 4), Rg1 (Figure 5) and Rg2 (Figure 6) can be rapidly excreted into bile when ginsenosides was given to rats (Cai et al., 2022). The elimination rate of protopanaxadiol ginsenosides such as ginsenoside Rb1 (Figure 7), Rd (Figure 8) and Rh2 (Figure 9) is slower than that of PPT ginsenosides (Li et al., 2015; Zhang et al., 2016; Shen et al., 2021). The pharmacokinetic properties of ginsenosides (ginsenoside Rg1, ginsenoside Rb1, ginsenoside Rc (Figure 10)) and aconitine alkaloids (benzoylmesaconine (Figure 11), aconitine (Figure 12)) in SFI showed a linear relationship in the dose range of 2–8 mL/kg (Zhang et al., 2016; Li S. et al., 2022).

Modern studies have shown that SFI is almost safe at conventional therapeutic doses, and the incidence of adverse reactions is relatively low (0.076%), such as rash, itching, nausea, vomiting, dizziness, abdominal pain, and palpitation (Wang Z. F. et al., 2017).

## 4 Pharmacological activities of SFI on CVDs

**FIGURE 13:**
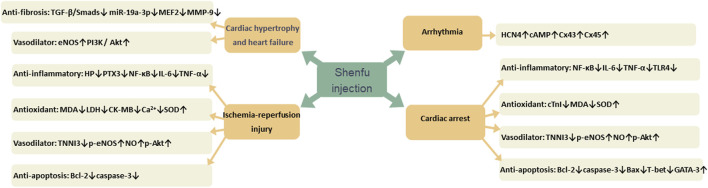
The pharmacological effect of Shenfu injection (SFI) on cardiovascular diseases.

Many studies have confirmed that SFI has therapeutic effects on a variety of CVDs, such as myocardial hypertrophy, heart failure, ischemia-reperfusion injury, cardiac arrest, and arrhythmia. Its mechanism of action is mainly related to reducing inflammation through NF-κB signaling pathway, oxidative stress by reducing free radical damage, dilating blood vessels by increasing nitric oxide (NO) content, decreasing fibrosis through TGF-𝛽/Smads signaling pathway and reducing apoptosis by increasing the expression of apoptosis proteins (Figure 13).

### 4.1 Cardiac hypertrophy and heart failure

Cardiac hypertrophy is mainly manifested as thickening of ventricular walls and an increase in cardiomyocyte size, closely related to cardiac fibrosis and heart failure (Feng et al., 2019). As time progresses and in settings of sustained stress, cardiac hypertrophy and fibrosis will eventually lead to heart failure (Gallo et al., 2019; Zhao D. et al., 2021; Methatham et al., 2021). Inhibiting cardiac hypertrophy and fibrosis is an effective way to treat heart failure.

TGF-𝛽/Smads plays a key role in the pathogenesis of myocardial fibrosis. The previous research suggested that TGF-𝛽1 bind to receptor, recruited and phosphorylated type I receptor, induced phosphorylation of Smad2 and Smad3. The phosphorylated Smad2 and Smad3 formed a trimer complex with Smad4. Then the complex transferred into the nucleus and regulated the transcription of target genes, regulating the synthesis of collagen fibers and the activation of fibroblasts. Smad7 can competitively bind to the type I receptor of TGF-𝛽1 and inhibit the signal transduction of TGF-𝛽1/Smads pathway (Stewart et al., 2018; Wang L. et al., 2021). Ni et al. (2017) found that SFI can effectively improve cardiac function in the rate model of congestive heart failure (CHF) and attenuate ventricular remodeling and myocardial fibrosis by regulating TGF-𝛽/Smads signaling pathway, upregulating Smad7 and downregulating TGF-𝛽1, Smad2 and Smad3 gene expression.

Inflammatory response is trigged in the myocardial infarction area which can result in cardiac remodeling and heart failure, accumulating high levels of monocytes and neutrophils (Halade and Lee, 2022). Inflammatory cells can also stimulate repair pathways, with increasing the content of extracellular matrix in the myocardium, including matrix metalloproteinase-9 (MMP-9) and collagen (Valiente-Alandi et al., 2018). SFI has anti-inflammatory effect, which can reduce the content of inflammatory factors such as tumor necrosis factor-α (TNF-α), interleukin-1𝛽 (IL-1𝛽) and interleukin-6 (IL-6) in serum of rats, and also reduce the expression of fibronectin, collagen I, collagen III and MMP-9 protein (Ni et al., 2017; Guo et al., 2022).

Endothelial function is essential for maintaining normal vasomotor function, and disruption of this function can result in vasomotor dysfunction (Sabe et al., 2022). When heart failure occurs, endothelial function is impaired, with vasoconstrictor substances increasing, and vasodilator substances decreasing (Monteiro et al., 2019). NO is the most famous vasodilator, while endothelin-1 (ET-1) is the most widely recognized vasoconstrictor (Miyauchi and Sakai, 2019). The production of NO mainly depends on the activity and quantity of endothelial nitric oxide synthase (eNOS). When the expression of eNOS mRNA increases, the number of eNOS synthesis increases. When eNOS binds with calmodulin (CaM), the activity of eNOS enchance, adversely, when binds with caveolin-1 (Cav-1), decrease. The phosphorylation of eNOS depends on the phosphatidylinositol 3-kinase/protein kinase B (PI3K-Akt) signaling pathway, which produces phosphorylated tyrosine residues, thereby provides an anchor site for the recruitment of PI3K to the membrane (Garcia and Sessa, 2019; Suvorava et al., 2022). Zhu et al. (2020) found that SFI can increase the expression of eNOS mRNA and CaM and promote NO synthesis, decrease the expression of Cav-1 and ET-1 content, promote eNOS phosphorylation via the PI3K/Akt signaling pathway.

MicroRNAs (miRNAs), encoded by myosin heavy chain (MHC) genes, are important regulatory factors of CVDs (Omidkhoda et al., 2019). MiR19a-3p plays an important role in cardiac hypertrophy. Myocytes specific enhancer factor 2A (MEF2A) is a target gene of miR-19a-3p and is highly expressed in cardiac hypertrophy while miR-19a-3p has low expression (Mao et al., 2018). SFI can upregulate the expression of miR-19a-3p, and downregulate the expression of MEF2A and 𝛽-myosin heavy chain (𝛽-MHC), so attenuate cardiac hypertrophy (Mao et al., 2018).

### 4.2 Ischemia-reperfusion injury

Myocardial ischemia-reperfusion injury is a significant factor that has a negative impact on the prognosis of myocardial infarction patients, causing myocardial stunning, no-reflow phenomena, reperfusion arrhythmia, and even permanent cardiomyocyte death. Therefore, it is critical to understand the mechanism of myocardial ischemia reperfusion and develop efficient treatments (Mokhtari-Zaer et al., 2018; Deng, 2021).

Apoptosis is involved in the pathogenesis of various CVDs and plays an important role in myocardial ischemia-reperfusion injury. Bcl-2 family proteins are important regulators of the process, prevent apoptosis by acting upstream of apoptosis proteins, such as caspase-3 and caspase-9 (King et al., 2023; Sahoo et al., 2023). Previous research found that SFI can upregulate the anti-apoptosis protein Bcl-2 and inhibit the consecutive activation of caspase-3 and caspase-9, both of which are intimately associated to apoptosis (Cao et al., 2005; Wang et al., 2009; Guo et al., 2016).

Oxidative stress is a risk factor for CVDs, and abnormally increased reactive oxygen species (ROS) is the main cause of oxidative stress. ROS combined with proteins and lipids damage cardiomyocytes (Peng et al., 2022). The superoxide dismutase (SOD) is the major antioxidant enzyme that degrade superoxide (Eleutherio et al., 2021). The glutathione system is widely recognized as one of the most potent endogenous antioxidant systems within cardiovascular system. Glutathione, one of the endogenous antioxidant molecules, can directly scavenge ROS caused by myocardial ischemia (Panday et al., 2020; Tan et al., 2023). Taurine is a common endogenous sulfur-containing amino acid with antioxidant activity and can inhibit the abnormal increase of ROS (Li et al., 2020). SFI dramatically decreased glutathione and taurine, increased SOD activity, and inhibited the rise in malondialdehyde (MDA), which is closely related to oxidative stress (Zheng et al., 2004; Cao et al., 2005; Wu et al., 2019).

Numerous studies have revealed that NO is a vasodilator with the ability to operate on cardiomyocytes and vascular endothelium via a variety of signaling pathways (Boycott et al., 2020; Cyr et al., 2020). The action of eNOS is primarily responsible for NO generation. NO generated by eNOS phosphorylation induces soluble guanylate cyclase (sGC) to create cyclic guanosine monophosphate (cGMP), a second messenger with cardiovascular protective properties (Mount et al., 2007; Zhang Q. et al., 2020; Lee et al., 2021). SFI activated eNOS phosphorylation via Akt, thereby promoting the production of NO (Wu et al., 2011; Wang et al., 2018).

### 4.3 Cardiac arrest

Cardiac arrest (CA), one of the leading causes of death, has a significant impact on the public health, particularly due to its persistent increase worldwide (Vazquez and Sudhir, 2023). Post-cardiac arrest syndrome (PCAS) is a group of diseases characterized by systemic ischemia/reperfusion injury, hypoxic brain injury and myocardial dysfunction after cardiac arrest (Jou et al., 2020). It is associated with cardiovascular ischemia/reperfusion injury and cardiovascular toxicity, including factors such as excessive activation of inflammatory cytokines and catecholamines (Lazzarin et al., 2022). Matrix metalloproteinases, tumor necrosis factor, and interleukins each have a special prognostic function in PCAS. High inflammatory cytokine levels have been linked to poor neurologic and/or death outcomes (Jou et al., 2020).

NF-κB signaling pathway is one of the important pathways regulating inflammation and plays a key regulatory role in the occurrence and development of various CVDs (Cheng et al., 2023). Study have reported that in cardiac arrest swine, SFIremarkedly decreased levels of many inflammatory cytokines, such as TNF-α, IL-6, mRNA and protein levels of myocardial TLR4 and NF-κB (Gu et al., 2021). TLR4, as a ‘portal’ protein, regulates the initiation of the inflammatory chain reaction of the body‘s immunity and mediates the inflammatory response (Fitzgerald and Kagan, 2020).

Na^+^-K^+^-ATPase enzyme and Ca^2+^-ATPase enzyme, ubiquitous enzymes in the heart, play a crucial role in process of CVDs (Fedosova et al., 2021). Na^+^-K^+^-ATPase transports two Na^+^ ions extrude out of the cell in exchange for one K^+^ ions, thereby maintaining the concentration gradients across the cell membrane (Fedosova et al., 2021; Obradovic et al., 2023). Ca^2+^-ATPase, a crucial role for cellular Ca^2+^ homeostasis, maintains normal intracellular calcium concentration and prevents calcium overload (Nguyen et al., 2023; Ye et al., 2023). It is reported that SFI increased Na^+^-K^-^ATPase and Ca^2+^-ATPase activity (Ji et al., 2011).

### 4.4 Arrhythmia

The normal electrical activity of the heart is initiated by special pacemaker cells located in the sinoatrial node (Liang et al., 2021). Dysfunction or loss of pacemaker cells can cause arrhythmia (Liu and Yuan, 2021b). The transplantation of stem cells is regarded as a kind of feasible treatment for arrhythmia (Sattayaprasert et al., 2020). It has been reported that bone marrow mesenchymal stem cells (BMSCs) with specific phenotypes can be transformed into pacemaker-like cells after special treatment (Chauveau et al., 2014). Moreover, HMSCs possess the capability to regulate arrhythmia substrates by altering their secretory groups in diseases (Sattayaprasert et al., 2020).

*In vitro*, SFI can activate inward pacemaker current of BMSCs in a concentration-dependent manner, increase HCN4 expression and cAMP content in BMSCs, induce BMSCs proliferation, promote their differentiation into pacemaker-like cells (Zhao X. et al., 2021). The HCN4 gene serves as the molecular basis for the pacemaker current, contributing significantly to inward current during depolarization and playing a crucial role in the generation and autonomous regulation of heart rate (Bucchi et al., 2012; D'Souza et al., 2021; Hoekstra et al., 2021). Bone marrow mesenchymal stem cells treated with SFI retained the function of sinoatrial node in rabbits with sinoatrial node syndrome, improved the expression of HCN4 gene and gap junction proteins (Cx43 and Cx45), and significantly upregulated the expression of cAMP in sinoatrial node (Chen Q. et al., 2022).

In addition, SFI has certain pharmacological effects on nervous system, respiratory system and digestive system. For example, SFI has a protective effect on lipopolysaccharide-induced septic shock in rabbits (Liu X. et al., 2019). It can reduce bile duct injury in rats with acute obstructive cholangitis (Tan et al., 2019) and increase the level of acetylcholine in acute liver injury in septic young rats (Wu et al., 2022). It also has a protective effect on lung and intestinal epithelial injury in mice with acute gastrointestinal injury (Zheng et al., 2022).

## 5 Clinical trial of SFI in CVDs

There are many clinical trials related to SFI, and nine clinical trials have been conducted to study its role in the treatment of CVDs. These clinical trials have demonstrated that SFI can improve cardiac function and corresponding indicators in patients with CVDs, including heart failure, myocardial infarction, cardiac arrest after resuscitation, coronary syndrome, coronary heart disease and other diseases (Table 1).

**TABLE 1:** Clinical trial of SFI in cardiovascular disease.

Disease	Number of patients	Dose of SFI (mL)	Duration	Route of administration	Outcome measures	References
acute heart failure	80 patients	50	7 days	i.v	cardiac function, clinical symptoms and quality of life	Wang et al. (2019b)
	55 patients	100	24 h	i.v	CI, cardiac output and stroke volume index	Li et al. (2022a)
Chronic heart failure	80 patients	50	7 days	i.v	LVEF, LVED, BNP,Fas, TNF-α, IL-6, mortality, readmission rate	Liu et al. (2015)
	171 patients	60	2 weeks	i.v	the all-cause mortality,6MWT	Wang et al. (2017a)
	55 patients	50	7 days	i.v	Cardiacfunction, LVEF, NT-proBNP, TNF-α, IL-6	Gao et al. (2021)
	91 patients	40	7 days	i.v	Cardiac function, LVEF, NT-proBNP, BNP	Guo et al. (2021)
coronary syndrome	74 patients	40	4–6 h	i.v	the level of NGAL in urine	Guo et al. (2017)
myocardial infarction	20 patients	80	5 days	i.v	the area of myocardial infarction	Wang et al. (2021b)
cardiac arrest	492 patients	100	28 days	i.v	28-day and 90-day survival rates, the mechanical ventilation time and hospitalization time	Zhang et al. (2017)

### 5.1 The effect of SFI in patients with acute heart failure

Infusion of SFI in 80 patients with acute heart failure can improve cardiac function, clinical symptoms and quality of life (Wang et al., 2019b). Fifty patients with acute decompensated heart failure were treated with combination therapy. Compared with simple infusion of levosimendan, the improvement of hemodynamic parameters including CI, cardiac output and stroke volume index was more significant, especially in patients with acute decompensated heart failure with hypotension (Li M. et al., 2022).

### 5.2 The effect of SFI in patients with chronic heart failure

SFI was used to treat 80 patients with acute exacerbation of chronic heart failure, which could improve the symptoms, quality of life, exercise tolerance, improve left ventricular ejective fraction (LVEF), reduce left ventricular end diastolic diameter (LVED), plasma brain natriuretic peptide (BNP) and cytokine Fas, TNF-α, IL-6 levels, reduce mortality and readmission rate (Liu et al., 2015). SFI was administered to 171 patients suffering from chronic heart failure on the basis of Western medicine. Compared with Western medicine alone, it could reduce the all-cause mortality by 30.99%, increase the 6-min walking distance (6MWT) and improve the quality of life (Wang X. et al., 2017). Patients with coronary heart disease complicated with chronic heart failure were treated with SFI and furosemide injection for 7 days. The effect was better than that of furosemide injection alone in improving cardiac function, LVEF, N-terminal B-type natriuretic peptide (NT-proBNP), TNF-α, IL-6 (Gao et al., 2021). For 7 days, SFI and sodium nitroprusside were administered intravenously to 91 patients who had coronary heart disease and chronic heart failure. The effect was better than that of sodium nitroprusside injection alone in improving cardiac function, LVEF, NT-proBNP and BNP (Guo et al., 2021).

### 5.3 The effect of SFI in patients with other CVDs

Infusion of SFI 1h before coronary angiography in 74 patients with coronary syndrome undergoing percutaneous coronary intervention (PCI) significantly reduced the level of neutrophil gelatinase-associated lipocalin (NGAL) in urine and effectively prevent contrast-induced acute kidney injury (Guo et al., 2017). SFI was used to treat patients with ST-segment elevation myocardial infarction before PCI and maintained for 5 days after PCI. Compared with patients treated with placebo, SFI reduced the area of myocardial infarction (Wang X. et al., 2021). A total of 492 cardiac arrest patients received bi-daily intravenous SFI infusions over a span of 28 days. The 28-day and 90-day survival rates were improved, the mechanical ventilation time and hospitalization time were shortened, and the recovery of spontaneous circulation after cardiac arrest was effectively improved (Zhang et al., 2017).

## 6 Concluding remarks and future perspectives

The results of numerous research studies in the past have demonstrated that SFI exerts varying degrees of therapeutic effects on various types of CVDs, such as heart failure, myocardial hypertrophy, myocardial ischemia, cardiac arrest, arrhythmia, and so forth. SFI plays a therapeutic role through multiple different targets, such as TGF-Smads, PI3K-Akt, eNOS-Akt pathway, and so on. SFI has been used in China for more than 30 years. It is a commonly used drug for clinical treatment of CVDs. No serious adverse reactions have been found so far.

In general, SFI is a promising drug for the treatment of CVDs. However, SFI has the characteristics of multi-component, multi-target and multi-pathway, which increases the difficulty of research. There is still a lack of in-depth study on the mechanism of SFI. In addition, large-scale, high-quality, multi-center clinical trials are needed to determine the comparison of SFI with traditional CVDs treatment regimens.
